# Association between Hemiarthroplasty vs. Total Hip Arthroplasty and Major Surgical Complications among Patients with Femoral Neck Fracture

**DOI:** 10.3390/jcm9103203

**Published:** 2020-10-03

**Authors:** Takahisa Ogawa, Toshitaka Yoshii, Mutsuko Moriwaki, Shingo Morishita, Yoto Oh, Kazumasa Miyatake, Ara Nazarian, Koichiro Shiba, Atsushi Okawa, Kiyohide Fushimi, Takeo Fujiwara

**Affiliations:** 1 Department of Orthopedic Surgery, Tokyo Medical and Dental University Graduate School of Medicine, Tokyo 113-8519, Japan; morsorth@tmd.ac.jp (S.M.); oh.orth@tmd.ac.jp (Y.O.); miyatake.orj@tmd.ac.jp (K.M.); okawa.orth@tmd.ac.jp (A.O.); 2 Department of Global Health Promotion, Tokyo Medical and Dental University Graduate School of Medicine, Tokyo 113-8519, Japan; fujiwara.hlth@tmd.ac.jp; 3 Center for Advanced Orthopaedic Studies, Department of Orthopaedic Surgery, Beth Israel Deaconess Medical Center, Harvard Medical School, Boston, MA 02215, USA; anazaria@bidmc.harvard.edu; 4 Department of Tokyo Metropolitan Health Policy Advisement, Graduate School of Medicine, Tokyo Medical and Dental University, Tokyo 113-8510, Japan; mmoriwaki.qmc@tmd.ac.jp; 5 Department of Epidemiology, Harvard T.H. Chan School of Public Health, Boston, MA 02115, USA; Shiba_k@g.harvard.edu; 6 Department of Health Policy and Informatics, Graduate School of Medicine, Tokyo Medical and Dental University, Tokyo 113-8510, Japan; kfushimi.hci@tmd.ac.jp

**Keywords:** Femoral neck fracture, total hip arthroplasty, hemiarthroplasty, national database, National Registration Database, complications, hip dislocation, geriatric fractures

## Abstract

Previous studies have shown better clinical outcomes after total hip arthroplasty (THA) compared to hemiarthroplasty (HA) for displaced femoral neck fracture. However, few studies have focused on the surgical risks of the two procedures. Therefore, we investigated the perioperative complications of HA and THA in femoral neck fracture, using a large nationwide inpatient database. A total of 286,269 patients (281,140 patients with HA and 5129 with THA) with a mean age of 81.7 were enrolled and HA and THA patients were matched by a propensity score to adjust for patient and hospital characteristics. Patients in a matched cohort were analyzed to compare complications and mortality. The systemic complication rate was not significantly different after a propensity score matching of 4967 pairs of patients. However, the incidence of both hip dislocation and revision surgery was more frequent in the THA group (Risk difference (RD), 2.74; 95% Confidence interval (CI), 2.21–3.27; *p* < 0.001; RD, 2.82; 95% CI, 2.27–3.37; *p* < 0.001, respectively). There was no significant difference in 30 day in-hospital mortality among the two groups. The risk of dislocation and reoperation was higher for THA than for HA in elderly patients with a femoral neck fracture in this retrospective study using a nationwide database.

## 1. Introduction

Femoral neck fracture is a representative trauma causing disability and morbidity, especially among elderly people. Open reduction and internal fixation often result in poor outcomes, and either hemiarthroplasty (HA) or total hip arthroplasty (THA) is recommended for displaced femoral neck fractures [1,2]. Previous studies have found that functionally demanding patients occasionally have problems after HA, such as pain, acetabular erosion, or implant loosening which, consequently, requires revision surgery. Recent studies have demonstrated the superior clinical outcomes of THA compared to HA [3,4,5,6,7,8,9,10]. However, the HA procedure is generally less extensive than the THA procedure. The average age of patients who require surgery also becomes higher, as the aging population increases, which could contribute to increased risk of surgical complications [11,12,13,14]. Therefore, when making decisions regarding surgical procedures, surgeons need to consider surgical extensiveness and complications in addition to functional outcomes.

Previous studies have reported that THA requires a greater amount of blood transfusion than HA [1,6,8,15] and that it could be associated with a higher incidence of perioperative complications [16,17]. However, other studies reported no differences in the complication rate between the two procedures [5,6]. It is difficult to obtain robust conclusions about surgical risks as most studies investigating femoral neck fracture are relatively small case series and have primarily investigated local complications. Some studies have evaluated systemic complications after surgery for displaced femoral neck fracture. However, those investigations did not match the baseline characteristics, such as age and health status, and thus the result between HA and THA patients could be biased. We aimed at comparing HA and THA in terms of perioperative complications, including local complications as well as systemic complications, using a large national inpatient database. Moreover, we performed a propensity score matching analysis to minimize confounding effects.

## 2. Materials and Methods

### 2.1. Data Source

We used the Japanese Diagnosis Procedure Combination inpatient database, which includes discharge abstracts and administrative claims data for 1549 acute care hospitals, covering 90% of all tertiary-care emergency hospitals in Japan. Patient baseline characteristics; main diagnoses for admission, activity of daily life score and comorbidities presented on admission, and complications after admission; surgical procedures coded with Japanese original codes; and length of hospital stay were the data contained in this database. Medical diagnosis, comorbidity, and complications were coded with the International Classification of Disease and Related Health Problems Tenth Revision codes (ICD-10) [18] (Table A1 and Table A2). A previous study validated the database of these diagnostic and procedural records with approximately 80% sensitivity and 90% specificity [19]. Approval for this study was obtained from the institutional review board (M2000-788). The requirement to obtain informed consent from individual patients was waived due to the anonymized data.

### 2.2. Study Population

The cohort comprised all consecutive patients hospitalized with a primary diagnosis of femoral neck fracture (ICD-10, S7200) who underwent surgery, either HA (HA, K0811; K-code, which is an originally developed national operation code) or THA (THA, K0821), between 1 April 2011 and 31 March 2018. Patients more than 60 of age were included.

### 2.3. Main Exposure

The surgical approaches were categorized into hemiarthroplasty (K0811) and THA (K0821).

### 2.4. Clinical Outcomes

We investigated 30 day in-hospital mortality and postoperative complications. Postoperative complications contained both systemic complications and local complications. The following complications—coronary heart disease, heart failure, respiratory disorders, pulmonary embolism, stroke, renal failure, urinary tract infection, sepsis, intensive care unit (ICU) admission, and blood transfusion—were included as systemic complications, and we defined revision surgery, hip dislocation, surgical site infection, and debridement procedure as local complications. We accessed revision surgery as a surgery coded as hemiarthroplasty (K0811) or total hip arthroplasty (K0821) after the HA or THA index. We also investigated anesthesia time, length of hospital stay, and daily average medical cost.

### 2.5. Covariates

Data on age, gender, body mass index (BMI), smoking history, comorbidities at admission, activity of daily life (ADL) score upon patient admission, hospital bed size, and annual hospital surgical volume for hip fracture surgery were evaluated. We defined hospital surgical volume as the annual number of patients undergoing hip fracture procedures at each hospital [20]. BMI was categorized as less than 18.5, from 18.5 to 24.9, 25.0 to 29.9, and more than 30.0 kg/m^2^. Congestive heart failure, peripheral vascular disease, myocardial infarction, heart failure, peripheral vascular disease, stroke, dementia, pulmonary disorders, peptic ulcer, mild, moderate, and severe disease, diabetes with or with complications, hemiplegia, renal failure, cancer, metastatic cancer, HIV, hypertension, stable angina, Parkinson’s disease, and anemia were the comorbidities that we evaluated upon admission based on the components of the Carlson Comorbidity Index [21]. The ADL assessment was performed by calculating the Barthel Index (BI). The BI is an ordinal scale that measures ADL performance and total BI is a cumulative score of these 10 items [22]. A maximum score of 100 corresponds to complete independence, and a minimum score of 0 corresponds to total dependence.

### 2.6. Statistical Analysis

Propensity score matching was used to identify a cohort of patients with similar baseline characteristics to account for differences in baseline characteristics between the two groups of eligible participants. A propensity score is the conditional probability of having a specific exposure such as HA versus THA on a set of baseline measured covariates [23,24]. We estimated the propensity score using a multivariable logistic-regression model, with THA as the dependent variable and all the baseline characteristics described in Table A3 as covariates. Then we matched patients in the HA and THA groups with a 1:1 matching protocol without replacement. We set caliper width for matching equal to 0.2 of the standard deviation of the logit of the propensity score. We estimated standardized differences for all the baseline covariates before and after matching to assess rematch baseline. Standardized differences of less than 10% for a given baseline covariates indicate relatively balanced matching [25]. For the comparative risk of other postoperative complications—anesthesia time, length of hospital stay, and daily average medical cost—we used a Generalized Estimating Equations(GEE)s and calculated percent absolute risk differences (% absolute risk difference (RD), with 95% confidence intervals (CI)). In the matched cohort, we plotted survival curves for revision surgery using the Kaplan–Meier method and compared patients undergoing HA vs. THA using the log-rank test. A Cox regression model with robust variance estimator was used to estimate the time to revision surgery during hospitalization. The type I error probability was set to 0.05 for all analyses. All statistical analyses were performed using Stata version 16.1 (Stata Corp, College Station, TX, USA).

### 2.7. Sensitivity Analyses

We conducted sensitivity analyses and used a stabilized inverse probability weight model, instead of propensity score matching, to account for confounding.

## 3. Results

### 3.1. Study Population

A total of 286,269 patients (mean (Standard Deviation(SD)) age, 81.7 years (8.40years)) who underwent arthroplasty, 281,140 (98.2%) who underwent HA, and 5129 (1.8%) who underwent THA, were included, of which 222,993 (77.9%) were women. Mean follow-up was 37.4 days. Compared to patients undergoing HA, patients undergoing THA were younger (HA, mean (SD) 81.8 (8.32) vs. THA, 73.6 (8.72); standardized mean difference (SMD), 95.9%) and had a higher BMI (25–30; 8.4% vs. 13.2%; SMD, 15.7%). They had fewer comorbidities such as heart failure (7.1% vs. 4.1%; SMD, 13.1%), hypertension (34.8% vs. 29.7%; SMD, 10.9%) history of stroke (10.4% vs. 5.9%; SMD, 16.2%), and dementia (16.5% vs. 7.1%; SMD, 29.5%; Table 1, Table A3). The THA group included patients with higher ADL scores (19.55 vs. 33.43; SMD, 43.8%) and longer waiting time (>72 hours) for surgery (50.8% vs. 66.4%; SMD, 32%) as well as more patients who underwent surgery at both large and teaching hospitals (8.2% vs. 14.6%; SMD, 20.2%; 2.8% vs. 10.3%; SMD, 30.5%, respectively) with general anesthesia (56.5% vs. 70.1%; SMD, 28.5%) than the HA group. Patients with osteoarthritis before hip fracture more likely underwent THA than HA (0.1% vs. 6.7%; SMD, 36.8%).

### 3.2. Outcomes after Matching

A total of 4967 pairs of patients undergoing HA and THA were obtained after propensity score matching. A standardized difference among two groups was less than 10% for all covariates and this suggests matching agreed achieved balance (Table 2). Patients undergoing THA were significantly associated with local complications (306 patients (6.2%) vs. 116 patients (2.3%); absolute risk difference, RD, 3.83; 95%CI, 3.04–4.61; *p* < 0.001) but not mortality (RD, 0.08; 95%CI, −0.24–0.4; *p* = 0.62) and systemic complications (RD, −0.26; 95%CI, −1.39–0.86; ˆ = 0.65) compared to patients undergoing HA, after the matching, as shown in Table 2 and Table 3.

Any systemic complication was observed in 10.0% of patients in the HA group, whereas the prevalence of systemic complication was 9.1% in the THA group; there was no significant difference between the two groups (RD, −0.26; 95%CI, −1.39–0.86; *p* = 0.65). However, blood transfusion and Intensive Care Unit (ICU) admission after surgery was more often found in the THA group than in the HA group (RD, 6.85; RD, 1.13; both *p* < 0.001).

**Figure 1:**
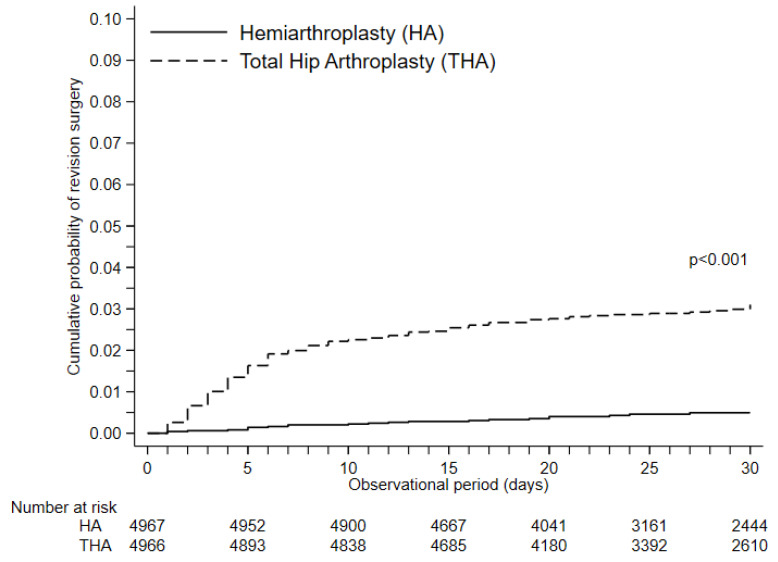
Kaplan–Meier curve of secondary revision surgery. The *p*-value of the log-lank test was <0.001.

Any local complication was observed in 2.3% of patients in the HA group, as opposed to 6.2% of patients in the THA group (RD, 3.83; 95%CI, 3.04–4.61; *p* < 0.001). The incidence of hip dislocation, surgical site infection, debridement, and revision surgery were significantly higher in THA group compared to the HA group (RD, 2.74; RD, 0.54; RD, 0.99; RD, 2.82, respectively, all *p* < 0.002). THA was significantly associated with revision surgery during hospital stay, compared to hemiarthroplasty (Hazard Ratio, 6.68; 95% confidence interval, 4.27−10.46; *p* < 0.001; Figure 1). THA required longer anesthesia time (RD, 37.17 minutes; 95%CI, 34.99–39.35; *p* < 0.001) than HA. The daily average medical cost was significantly higher in the THA group than in the HA group before and after matching (RD, 7.71 dollars; 95%CI, 6.79–8.63; *p* < 0.001). The result of the sensitivity analysis shows a similar association between surgical procedure, such as THA or HA, and complications (Table A4).

## 4. Discussion

The present study showed that the incidence of local complications in surgically treated femoral neck fractures were significantly higher among THA group compared to the HA group after propensity score matching (RD, 3.83; *p* < 0.001). The overall systemic complication rate was 9.2%, and mortality was 0.6%. Neither the systemic complication rate nor the mortality rate differed between the two groups when comparing HA and THA, which is consistent with the findings of previous studies [7,8,26,27].

The THA group showed a higher incidence of local complications including surgical site infection, debridement, and revision after surgery than the HA group. Dislocation is the major concern after both HA and THA for displaced femoral neck fractures in elderly patients. Previous studies have shown that the postoperative dislocation rate is higher in THA than in HA [28,29], whereas other studies have indicated no increase in the complication rate, including dislocation rate, after THA [6,7]. Our results showed that the risk of hip dislocation during the postoperative in-hospital period was significantly higher in the THA group than in the HA group dislocation (RD, 2.74%; 95%CI, 2.21–3.27; *p* < 0.001) which we supposed to be the main cause of the higher risk of revision surgery in the THA group (RD, 2.82; 95%CI, 2.27–3.37; *p* < 0.001). The reason for this higher risk of dislocation with THA may be the smaller size of the head of the femoral implant in THA than in HA. Previous studies have demonstrated that the size of the femoral head affects the incidence of dislocation after surgery [30,31,32]. Another potential reason for this higher risk of dislocation with THA is that a large preoperative range of motion and soft tissue damage due to trauma may contribute to high risk of dislocation [33,34,35,36]. Hip dislocation within five weeks after the surgery, especially, is a risk factor of revision surgery compared to delayed dislocation [37]. Extensive care should be taken to prevent dislocation during hospitalization after THA in elderly patients with femoral neck fracture.

The results of this study show longer anesthesia time and higher rate of blood transfusion with THA than with HA, suggesting greater surgical extensiveness and complicated procedures of THA for elderly patients, which is consistent with previous studies [1,6,8,15,38]. Longer surgical time and blood transfusion are also associated with surgical site complication or following a debridement procedure [39,40,41,42]. These complications could also be attributed to the higher incidence of revision surgery in addition to hip dislocation.

The results of our study showed that daily average medical costs for hospitalization were significantly higher in the THA group compared to the HA group. Although systemic complication rates were similar in the two groups in this study, the expense of THA was higher in the early period after surgery, possibly because of additional implants (i.e., acetabular component) and additional blood transfusion, as previously reported [1,43]. Furthermore, the higher incidence of dislocation and subsequent reoperation may have contributed to the higher medical costs in the THA group. Nonetheless, the Quality of Life (QOL) of patients is reported to be better after THA than after HA, and the difference becomes increasingly marked over time [6,7]. Therefore, the cost-effectiveness of the THA procedure may become more favorable after long-term follow-up.

The present study has some limitations. First, the national database did not include detailed information about surgical procedures or implants. Therefore, several important factors regarding dislocation, such as type of surgical approach and type and size of the implant, and angle of implant placement were not evaluated. Second, information about each surgeon’s skills, experience, and operating time was not available. Surgical skills are known to be associated with fewer complications [20,44,45]. However, we included hospital procedure volume in the statistical model to consider the confounding effect of the surgeon’s skills. In addition, the similar result of our sensitivity analysis supports the robustness of our research. We used hospital procedure volume as a proxy, as experienced hip expertise surgeons are likely allocated to large-volume hospitals. Third, we matched various factors, including pre- and perioperative comorbidities and ADL scores, among others. However, the degree of these comorbidities and functional hip score could not be obtained and were not matched. These factors can be residual confounding factors even after propensity score matching.

## 5. Conclusions

Compared to HA in elderly patients with femoral neck fracture, THA was associated with a higher incidence of local complications and reoperation. Surgeons should consider the merits and demerits of both procedures when deciding on the surgical method for treating femoral neck fracture in elderly patients.

## 6. Patents

None.

## Figures and Tables

**Table 1:** Patient characteristics.

	Before Matching			After Matching		
	Hemiarthroplasty	Total Hip Arthroplasty	SMD	Hemiarthroplasty	Total Hip Arthroplasty	SMD
	*N* = 281,140	*N* = 5129	%	*N* = 4967	*N* = 4967	%
Age, years	81.80 (8.32)	73.63 (8.72)	95.9	74.53 (8.95)	73.69 (8.74)	9.5
Age						
60-	27,334 (9.7)	2048 (39.9)	74.6	1954 (39.3)	1965 (39.6)	0.5
70-	71,128 (25.3)	693 (33.0)	17	1585 (31.9)	1646 (33.1)	2.6
80-	133,167 (47.4)	1169 (22.8)	53.3	1175 (23.7)	1140 (23.0)	1.7
90-	48,261 (17.2)	217 (4.2)	42.8	250 (5.0)	214 (4.3)	3.4
100-	1250 (0.4)	2 (0.0)	8.3	3 (0.1)	2 (0.0)	0.9
Female	219,042 (77.9)	3951 (77.0)	2.1	3829 (77.1)	3826 (77.0)	0.1
BMI						
<18.5	75,851 (27.0)	1055 (20.6)	15.1	1068 (21.5)	1032 (20.8)	1.8
18.5–25	157,959 (56.2)	2994 (58.4)	4.4	2872 (57.8)	2893 (58.2)	0.9
25–30	23,574 (8.4)	679 (13.2)	15.7	623 (12.5)	650 (13.1)	1.6
≥30	2953 (1.1)	101 (2.0)	7.5	94 (1.9)	96 (1.9)	0.3
Missing	20,803 (7.4)	300 (5.8)	6.2	310 (6.2)	296 (6.0)	1.2
Smoking						
Never-smoking	225,634 (80.3)	3847 (75.0)	12.6	3726 (75.0)	3711 (74.7)	0.7
Smoking	31,669 (11.3)	774 (15.1)	11.3	762 (15.3)	758 (15.3)	0.2
Missing	23,837 (8.5)	508 (9.9)	4.9	479 (9.6)	498 (10.0)	1.3
Hip Osteoarthritis	390 (0.1)	346 (6.7)	36.8	235 (4.7)	194 (3.9)	4.1
Coronary heart disease	3785 (1.3)	53 (1.0)	2.9	50 (1.0)	50 (1.0)	0
Heart failure	19,996 (7.1)	211 (4.1)	13.1	216 (4.3)	205 (4.1)	1.1
Peripheral vascular disease	1384 (0.5)	20 (0.4)	1.5	17 (0.3)	19 (0.4)	0.7
Stroke	29,152 (10.4)	305 (5.9)	16.2	316 (6.4)	301 (6.1)	1.3
Dementia	46,326 (16.5)	363 (7.1)	29.5	430 (8.7)	359 (7.2)	5.3
Pulmonary Disorders	10,761 (3.8)	140 (2.7)	6.2	116 (2.3)	135 (2.7)	2.4
Connective tissue disease	725 (0.3)	27 (0.5)	4.3	38 (0.8)	27 (0.5)	2.7
Peptic ulcer	8509 (3.0)	164 (3.2)	1	153 (3.1)	160 (3.2)	0.8
Mild liver disease	1522 (0.5)	19 (0.4)	2.5	22 (0.4)	19 (0.4)	0.9
Mild to moderate diabetes	42,034 (15.0)	784 (15.3)	0.9	774 (15.6)	764 (15.4)	0.6
Diabetes with complications	7337 (2.6)	135 (2.6)	0.1	122 (2.5)	134 (2.7)	1.5
Hemiplegia	18 (0.0)	0 (0.0)	1.1	0 (0.0)	0 (0.0)	0
Renal failure	13,073 (4.6)	196 (3.8)	4.1	210 (4.2)	194 (3.9)	1.6
Cancer	13,742 (4.9)	211 (4.1)	3.7	235 (4.7)	206 (4.1)	2.8
Metastatic cancer	1307 (0.5)	13 (0.3)	3.5	14 (0.3)	13 (0.3)	0.4
Sever liver disease	54 (0.0)	0 (0.0)	2	0 (0.0)	0 (0.0)	0
HIV	21 (0.0)	1 (0.0)	1	0 (0.0)	1 (0.0)	2
Hypertension	97,910 (34.8)	1525 (29.7)	10.9	1420 (28.6)	1489 (30.0)	3.1
Stable angina	19,813 (7.0)	276 (5.4)	6.9	259 (5.2)	271 (5.5)	1.1
Parkinson disease	8036 (2.9)	128 (2.5)	2.2	132 (2.7)	126 (2.5)	0.8
Anemia	8069 (2.9)	223 (4.3)	7.9	186 (3.7)	212 (4.3)	2.7
Barthel Index	19.55 (0.59)	33.43 (0.54)	43.8	32.16 (0.53)	32.90 (0.54)	2.1
Wait time for surgery						
Less than 72 hours	138,190 (49.2)	1723 (33.6)	32	1721 (34.6)	1681 (33.8)	1.7
More than 72 hours	142,950 (50.8)	3406 (66.4)	32	3246 (65.4)	3286 (66.2)	1.7
Anesthesia						
General Anesthesia	158,846 (56.5)	3595 (70.1)	28.5	3352 (67.5)	3504 (70.5)	6.6
Spinal Anesthesia	111,120 (39.5)	533 (10.4)	71.5	660 (13.3)	532 (10.7)	7.9
General and Epidural	9975 (3.5)	963 (18.8)	49.8	916 (18.4)	893 (18.0)	1.2
Spinal and Epidural	1199 (0.4)	38 (0.7)	4.1	39 (0.8)	38 (0.8)	0.2
Teaching Hospitals	8003 (2.8)	529 (10.3)	30.5	582 (11.7)	504 (10.1)	5
Hospital Volume, bed						
<100	49,585 (17.6)	599 (11.7)	16.9	612 (12.3)	583 (11.7)	1.8
100–200	87,074 (31.0)	1,437 (28.0)	6.5	1,321 (26.6)	1388 (27.9)	3
200–300	65,345 (23.2)	1,177 (22.9)	0.7	1,135 (22.9)	1145 (23.1)	0.5
300–400	35,514 (12.6)	644 (12.6)	0.2	622 (12.5)	623 (12.5)	0.1
400–500	20,552 (7.3)	523 (10.2)	10.2	516 (10.4)	509 (10.2)	0.5
>500	23,070 (8.2)	749 (14.6)	20.2	761 (15.3)	719 (14.5)	2.4
Annual Procedure Volume						
~30 case/year	19,504 (6.9)	506 (9.9)	10.6	582 (11.7)	488 (9.8)	6.1
30~100 case/year	123,221 (43.8)	2377 (46.3)	5.1	2259 (45.5)	2302 (46.3)	1.7
100~ case/year	138,415 (49.2)	2246 (43.8)	10.9	2126 (42.8)	2177 (43.8)	2.1

The values are given as the number of patients, with the percentage of the cohort in parentheses, with the exception of age, and Barthel Index, which are given as the mean and standard deviation. BMI, body mass index; HIV, human immunodeficiency virus; *N*, number of patients; SMD, standardized mean difference.

**Table 2:** Postoperative complications between the propensity score matched groups.

	Hemiarthroplasty	Total Hip Arthroplasty	Absolute Risk Difference (95% Confidence Interval (CI)), %	
	(*N* = 4967)	(*N* = 4967)		
	*N* (%)	*N* (%)		*p*-Value
Systemic complications	464 (9.3)	451 (9.1)	−0.26 (−1.39–0.86)	0.65
Local complications	116 (2.3)	306 (6.2)	3.83 (3.04–4.61)	<0.001
Systemic complications				
Coronary Heart Disease	62 (1.2)	50 (1.0)	−0.24 (−0.66–0.17)	0.25
Heart Failure	46 (0.9)	38 (0.8)	−0.16 (−0.51–0.19)	0.37
Respiratory Disorders	102 (2.1)	78 (1.6)	−0.48 (−1–0.04)	0.07
Pulmonary Embolism	19 (0.4)	23 (0.5)	0.08 (−0.18–0.34)	0.54
Stroke	28 (0.6)	20 (0.4)	−0.16 (−0.43–0.11)	0.25
Renal Failure	16 (0.3)	18 (0.4)	0.04 (−0.19–0.27)	0.73
Urinary Tract Infection	109 (2.2)	80 (1.6)	−0.58 (−1.12–−0.04)	0.034
Sepsis	11 (0.2)	18 (0.4)	0.14 (−0.07–0.35)	0.19
Local complications				
Hip dislocation	24 (0.5)	160 (3.2)	2.74 (2.21–3.27)	<0.001
Surgical site infection	47 (0.9)	74 (1.5)	0.54 (0.11–0.98)	0.014
Secondary revision surgery	30 (0.6)	170 (3.4)	2.82 (2.27–3.37)	<0.001

**Table 3:** Thirty-day mortality and other clinical outcomes between propensity score matched group.

	Hazard Ratio (95% CI)	*p*-Value		
Secondary revision surgery	6.68 (4.27–10.46)	<0.001		
	*N* (%)	*N* (%)	Absolute risk difference (95% CI), %	*p*-value
30-day Hospital Death	30 (0.6)	34 (0.7)	0.08 (-0.24–0.4)	0.62
Intensive Care Unit (ICU) admission	115 (2.3)	171 (3.4)	1.13 (0.47–1.79)	<0.001
Blood Transfusion	434 (8.7)	774 (15.6)	6.85 (5.58–8.11)	<0.001
	Mean (Standard Deviation(SD))	Mean (SD)	Absolute mean difference (95% CI), %	*p*-value
Anesthesia time, minute	110.31 (45.31)	146.92 (64.40)	36.61 (34.44–38.78)	<0.001
Length of hospital stay, day	35.93 (22.98)	38.88 (27.40)	2.96 (1.96–3.95)	<0.001
Daily average medical cost, USD	58.98 (21.24)	66.69 (25.28)	-7.71 (6.79–8.64)	<0.001

## Appendix A

**Table A1:** International Classification of Diseases (ICD)-10 definitions for comorbidities and complications.

Comorbidities	ICD-10 Codes
myocardial infarction	I21, I22, I252
heart failure	I50
peripheral vascular disease	I71, I790, I739, R02, Z958, Z959
cerebrovascular disease	I60-63, I65-66, G450-452, G458-459, G46, I64, G454, I670-672, I674, I675-679, I681-682, I688, I69
dementia	F00-03, F051, G30
pulmonary disease	J40-47, J60-67
connective tissue disease	M32, M34, M332, M053, M058-060, M063, M069, M050, M052, M051, M353
gastric ulcer	K25-28
mild liver disease	K702-703, K73, K717, K740, K742-746
diabetes mellitus	E109, E119, E139, E149, E101, E111, E131, E141, E105, E115, E135, E145
diabetes with complications	E102, E112, E132, E142, E142, E113, E133, E143, E104, E114, E117, E134, E144, E117
paralysis	G041, G820, G821, G822
renal failure	N03, N052-056, N072-074, N01, N18-19, N25
cancer	C0-9
metastatic cancer	C77-80
severe liver disease	K729, K766-7, K721
HIV	B20-24
anemia	D509
hypertension	I10
osteoarthritis of hip	M16
Complications	ICD-10 codes
surgical site infection	T793, T814
Debridement	K1000

**Table A2:** ICD-10 definitions for complications.

Complications	ICD-10 Codes
surgical site infection	T793, T814
acute coronary syndrome	I20-25
heart failure	I50
respiratory disorder	J12-18, J95-96
sepsis	A40-41, D65
pulmonary embolism	I26
cerebrovascular disease	I60-64
renal failure	N17-19
urinary tract infection	N390
Hip dislocation	K0631

**Table A3:** Patient characteristics (Full description).

	Before Matching			After Matching		
	Hemiarthroplasty	Total Hip Arthroplasty	SMD	Hemiarthroplasty	Total Hip Arthroplasty	SMD
	*N* = 281,140	*N* = 5129	%	*N* = 4967	*N* = 4967	%
Age	81.80 (8.32)	73.63 (8.72)	95.9	74.53 (8.95)	73.69 (8.74)	9.5
Age						
60-	27,334 (9.7)	2048 (39.9)	74.6	1954 (39.3)	1965 (39.6)	0.5
70-	71,128 (25.3)	1693 (33.0)	17	1585 (31.9)	1646 (33.1)	2.6
80-	133,167 (47.4)	1169 (22.8)	53.3	1175 (23.7)	1140 (23.0)	1.7
90-	48,261 (17.2)	217 (4.2)	42.8	250 (5.0)	214 (4.3)	3.4
100-	1250 (0.4)	2 (0.0)	8.3	3 (0.1)	2 (0.0)	0.9
Female	219,042 (77.9)	3951 (77.0)	2.1	3829 (77.1)	3826 (77.0)	0.1
BMI						
<18.5	75,851 (27.0)	1055 (20.6)	15.1	1068 (21.5)	1032 (20.8)	1.8
18.5–25	157,959 (56.2)	2994 (58.4)	4.4	2872 (57.8)	2893 (58.2)	0.9
25–30	23,574 (8.4)	679 (13.2)	15.7	623 (12.5)	650 (13.1)	1.6
≥30	2953 (1.1)	101 (2.0)	7.5	94 (1.9)	96 (1.9)	0.3
Missing	20,803 (7.4)	300 (5.8)	6.2	310 (6.2)	296 (6.0)	1.2
Smoking						
Never-smoking	225,634 (80.3)	3847 (75.0)	12.6	3726 (75.0)	3711 (74.7)	0.7
Smoking	31,669 (11.3)	774 (15.1)	11.3	762 (15.3)	758 (15.3)	0.2
Missing	23,837 (8.5)	508 (9.9)	4.9	479 (9.6)	498 (10.0)	1.3
Hip Osteoarthritis	390 (0.1)	346 (6.7)	36.8	235 (4.7)	194 (3.9)	4.1
Coronary heart disease	3785 (1.3)	53 (1.0)	2.9	50 (1.0)	50 (1.0)	0
Heart failure	19,996 (7.1)	211 (4.1)	13.1	216 (4.3)	205 (4.1)	1.1
Peripheral vascular disease	1384 (0.5)	20 (0.4)	1.5	17 (0.3)	19 (0.4)	0.7
Stroke	29,152 (10.4)	305 (5.9)	16.2	316 (6.4)	301 (6.1)	1.3
Dementia	46,326 (16.5)	363 (7.1)	29.5	430 (8.7)	359 (7.2)	5.3
Pulmonary Disorders	10,761 (3.8)	140 (2.7)	6.2	116 (2.3)	135 (2.7)	2.4
Connective tissue disease	725 (0.3)	27 (0.5)	4.3	38 (0.8)	27 (0.5)	2.7
Peptic ulcer	8509 (3.0)	164 (3.2)	1	153 (3.1)	160 (3.2)	0.8
Mild liver disease	1522 (0.5)	19 (0.4)	2.5	22 (0.4)	19 (0.4)	0.9
Mild to moderate diabetes	42,034 (15.0)	784 (15.3)	0.9	774 (15.6)	764 (15.4)	0.6
Diabetes with complications	7337 (2.6)	135 (2.6)	0.1	122 (2.5)	134 (2.7)	1.5
Hemiplegia	18 (0.0)	0 (0.0)	1.1	0 (0.0)	0 (0.0)	0
Renal failure	13,073 (4.6)	196 (3.8)	4.1	210 (4.2)	194 (3.9)	1.6
Cancer	13,742 (4.9)	211 (4.1)	3.7	235 (4.7)	206 (4.1)	2.8
Metastatic cancer	1307 (0.5)	13 (0.3)	3.5	14 (0.3)	13 (0.3)	0.4
Sever liver disease	54 (0.0)	0 (0.0)	2	0 (0.0)	0 (0.0)	0
HIV	21 (0.0)	1 (0.0)	1	0 (0.0)	1 (0.0)	2
Hypertension	97,910 (34.8)	1525 (29.7)	10.9	1420 (28.6)	1489 (30.0)	3.1
Stable angina	19,813 (7.0)	276 (5.4)	6.9	259 (5.2)	271 (5.5)	1.1
Parkinson disease	8036 (2.9)	128 (2.5)	2.2	132 (2.7)	126 (2.5)	0.8
Anemia	8069 (2.9)	223 (4.3)	7.9	186 (3.7)	212 (4.3)	2.7
Feeding						
0	79,240 (28.2)	881 (17.2)	26.5	893 (18.0)	862 (17.4)	1.6
5	115,601 (41.1)	1862 (36.3)	9.9	1794 (36.1)	1810 (36.4)	0.7
10	77,322 (27.5)	2269 (44.2)	35.4	2176 (43.8)	2178 (43.8)	0.1
Missing	8977 (3.2)	117 (2.3)	5.6	104 (2.1)	117 (2.4)	1.8
Chair/bed transfers						
0	201,968 (71.8)	2976 (58.0)	29.3	2820 (56.8)	2908 (58.5)	3.6
5	24,036 (8.5)	451 (8.8)	0.9	461 (9.3)	438 (8.8)	1.6
10	23,615 (8.4)	719 (14.0)	17.9	708 (14.3)	683 (13.8)	1.5
15	21,656 (7.7)	819 (16.0)	25.8	833 (16.8)	777 (15.6)	3.1
Missing	9865 (3.5)	164 (3.2)	1.7	145 (2.9)	161 (3.2)	1.9
Personal hygiene						
0	230,491 (82.0)	3505 (68.3)	32	3392 (68.3)	3417 (68.8)	1.1
5	41,256 (14.7)	1498 (29.2)	35.7	1471 (29.6)	1427 (28.7)	1.9
Missing	9393 (3.3)	126 (2.5)	5.3	104 (2.1)	123 (2.5)	2.6
Toilet						
0	220,017 (78.3)	3,213 (62.6)	34.7	3070 (61.8)	3141 (63.2)	3
5	29,787 (10.6)	880 (17.2)	19.1	883 (17.8)	838 (16.9)	2.4
10	22,752 (8.1)	907 (17.7)	28.9	905 (18.2)	859 (17.3)	2.4
Missing	8584 (3.1)	129 (2.5)	3.3	109 (2.2)	129 (2.6)	2.6
Bathing self						
0	231,390 (82.3)	3780 (73.7)	20.9	3623 (72.9)	3682 (74.1)	2.7
5	19,402 (6.9)	787 (15.3)	27.1	785 (15.8)	745 (15.0)	2.2
Missing	30,348 (10.8)	562 (11.0)	0.5	559 (11.3)	540 (10.9)	1.2
Ambulation						
0	216,824 (77.1)	3437 (67.0)	22.7	3303 (66.5)	3348 (67.4)	1.9
5	8293 (2.9)	296 (5.8)	13.8	314 (6.3)	284 (5.7)	2.5
10	9337 (3.3)	245 (4.8)	7.4	226 (4.6)	236 (4.8)	1
15	19,314 (6.9)	723 (14.1)	23.8	738 (14.9)	687 (13.8)	2.9
Missing	27,372 (9.7)	428 (8.3)	4.9	386 (7.8)	412 (8.3)	1.9
Stair climbing						
0	218,388 (77.7)	3583 (69.9)	17.9	3464 (69.7)	3489 (70.2)	1.1
5	10,304 (3.7)	284 (5.5)	8.9	272 (5.5)	275 (5.5)	0.3
10	17,565 (6.2)	665 (13.0)	22.9	693 (14.0)	635 (12.8)	3.4
Missing	34,883 (12.4)	597 (11.6)	2.4	538 (10.8)	568 (11.4)	1.9
Dressing						
0	213,137 (75.8)	3023 (58.9)	36.6	2905 (58.5)	2959 (59.6)	2.2
5	39,876 (14.2)	1151 (22.4)	21.5	1134 (22.8)	1101 (22.2)	1.6
10	21,980 (7.8)	858 (16.7)	27.4	850 (17.1)	814 (16.4)	1.9
Missing	6147 (2.2)	97 (1.9)	2.1	78 (1.6)	93 (1.9)	2.3
Bladder control						
0	179,341 (63.8)	2361 (46.0)	36.3	2337 (47.1)	2317 (46.6)	0.8
5	31,870 (11.3)	649 (12.7)	4.1	643 (12.9)	627 (12.6)	1
10	56,123 (20.0)	1890 (36.8)	38.1	1792 (36.1)	1801 (36.3)	0.4
Missing	13,806 (4.9)	229 (4.5)	2.1	195 (3.9)	222 (4.5)	2.7
Bowel control						
0	182,952 (65.1)	2444 (47.7)	35.7	2395 (48.2)	2398 (48.3)	0.1
5	31,251 (11.1)	622 (12.1)	3.2	614 (12.4)	601 (12.1)	0.8
10	53,424 (19.0)	1831 (35.7)	38.1	1755 (35.3)	1744 (35.1)	0.5
Missing	13,513 (4.8)	232 (4.5)	1.3	203 (4.1)	224 (4.5)	2.1
Wait time for surgery						
Less than 72 hours	138,190 (49.2)	1723 (33.6)	32	1721 (34.6)	1681 (33.8)	1.7
More than 72 hours	142,950 (50.8)	3406 (66.4)	32	3246 (65.4)	3286 (66.2)	1.7
Anesthesia						
General Anesthesia	158,846 (56.5)	3595 (70.1)	28.5	3352 (67.5)	3504 (70.5)	6.6
Spinal Anesthesia	111,120 (39.5)	533 (10.4)	71.5	660 (13.3)	532 (10.7)	7.9
General and Epidural	9975 (3.5)	963 (18.8)	49.8	916 (18.4)	893 (18.0)	1.2
Spinal and Epidural	1199 (0.4)	38 (0.7)	4.1	39 (0.8)	38 (0.8)	0.2
Teaching Hospitals	8003 (2.8)	529 (10.3)	30.5	582 (11.7)	504 (10.1)	5
Hospital Volume, bed						
<100	49,585 (17.6)	599 (11.7)	16.9	612 (12.3)	583 (11.7)	1.8
100–200	87,074 (31.0)	1437 (28.0)	6.5	1321 (26.6)	1388 (27.9)	3
200–300	65,345 (23.2)	1177 (22.9)	0.7	1135 (22.9)	1145 (23.1)	0.5
300–400	35,514 (12.6)	644 (12.6)	0.2	622 (12.5)	623 (12.5)	0.1
400–500	20,552 (7.3)	523 (10.2)	10.2	516 (10.4)	509 (10.2)	0.5
>500	23,070 (8.2)	749 (14.6)	20.2	761 (15.3)	719 (14.5)	2.4
Annual Procedure Volume						
~30 case/year	19,504 (6.9)	506 (9.9)	10.6	582 (11.7)	488 (9.8)	6.1
30~100 case/year	123,221 (43.8)	2377 (46.3)	5.1	2259 (45.5)	2302 (46.3)	1.7
100~ case/year	138,415 (49.2)	2246 (43.8)	10.9	2126 (42.8)	2177 (43.8)	2.1
Year						
2010	18,673 (6.6)	154 (3.0)	17	160 (3.2)	152 (3.1)	0.9
2011	19,917 (7.1)	192 (3.7)	14.8	192 (3.9)	187 (3.8)	0.5
2012	26,786 (9.5)	279 (5.4)	15.6	270 (5.4)	272 (5.5)	0.2
2013	28,549 (10.2)	364 (7.1)	10.9	373 (7.5)	352 (7.1)	1.6
2014	37,737 (13.4)	544 (10.6)	8.7	494 (9.9)	523 (10.5)	1.9
2015	38,091 (13.5)	674 (13.1)	1.2	648 (13.0)	661 (13.3)	0.8
2016	39,869 (14.2)	842 (16.4)	6.2	832 (16.8)	821 (16.5)	0.6
2017	38,810 (13.8)	964 (18.8)	13.5	885 (17.8)	931 (18.7)	2.4
2018	32,708 (11.6)	1116 (21.8)	27.4	1113 (22.4)	1068 (21.5)	2.2
Season						
Spring	64,476 (22.9)	1219 (23.8)	2	1194 (24.0)	1178 (23.7)	0.8
Summer	64,527 (23.0)	1111 (21.7)	3.1	1086 (21.9)	1078 (21.7)	0.4
Fall	77,139 (27.4)	1408 (27.5)	0	1328 (26.7)	1368 (27.5)	1.8
Winter	74,998 (26.7)	1391 (27.1)	1	1359 (27.4)	1343 (27.0)	0.7
Admission day of the week						
Sunday	26,262 (9.3)	502 (9.8)	1.5	491 (9.9)	489 (9.8)	0.1
Monday	49,885 (17.7)	939 (18.3)	1.5	878 (17.7)	914 (18.4)	1.9
Tuesday	45,088 (16.0)	812 (15.8)	0.6	802 (16.1)	786 (15.8)	0.9
Wednesday	42,041 (15.0)	825 (16.1)	3.1	787 (15.8)	796 (16.0)	0.5
Thursday	41,598 (14.8)	720 (14.0)	2.2	736 (14.8)	702 (14.1)	1.9
Friday	43,975 (15.6)	780 (15.2)	1.2	757 (15.2)	753 (15.2)	0.2
Saturday	32,291 (11.5)	551 (10.7)	2.4	516 (10.4)	527 (10.6)	0.7

The values are given as the number of patients, with the percentage of the cohort in parentheses, with the exception of age, and Barthel Index, which are given as the mean and standard deviation. BMI, body mass index; HIV, human immunodeficiency virus; *N*, number of patients; SMD, standardized mean difference.

**Table A4:** Sensitivity analysis (Stabilized Inverse Probability Weighting).

	Absolute Risk Difference (95% Confidence Interval(CI)), %a	*p*-Value
In-Hospital death	0.07 (−0.86–0.99)	0.89
Systemic complication	−0.02 (−2.17–2.12)	<0.001
Coronary Heart Disease	−0.81 (−1.2–−0.42)	<0.001
Heart Failure	0.35 (−0.71–1.41)	0.51
Respiratory Disorders	−0.47 (−1.28–0.34)	0.25
Pulmonary Embolism	−0.07 (−0.32–0.17)	0.55
Stroke	−0.3 (−0.6–0)	0.048
Renal Failure	0.11 (−0.43–0.64)	0.7
Urinary Tract Infection	−0.99 (−1.61–−0.37)	0.002
Sepsis	−0.14 (−0.37–0.09)	0.25
Intensive Care Unit (ICU) admission	1.91 (0.43–3.39)	0.012
Blood Transfusion	9.25 (5.62–12.88)	<0.001
Local complications	4.16 (2.48–5.83)	<0.001
Secondary revision surgery	4.4 (2.58–6.22)	<0.001
Hip dislocation	3.58 (2.06–5.11)	<0.001
Surgical site infection	0.33 (−0.23–0.89)	0.25
Debridement	0.42 (−0.07–0.92)	0.09
	Absolute risk difference (95% CI), %a	*p*-value
Anesthesia time	37.821	<0.001
Length of hospital stay	6.928	0.001
